# Impact of Integrating Rabies Education Into the Curriculum of Public Elementary Schools in Ilocos Norte, Philippines on Rabies Knowledge, and Animal Bite Incidence

**DOI:** 10.3389/fpubh.2019.00119

**Published:** 2019-05-24

**Authors:** Anna Charinna B. Amparo, Eunice Charis B. Mendoza, Dianne A. Licuan, Loida M. Valenzuela, Joye D. Madalipay, Sarah I. Jayme, Louise H. Taylor

**Affiliations:** ^1^Global Alliance for Rabies Control, Santa Rosa City, Philippines; ^2^Provincial Veterinary Office, Laoag City, Philippines; ^3^Department of Education, Ilocos Norte School Division, Laoag City, Philippines; ^4^Global Alliance for Rabies Control, Manhattan, KS, United States

**Keywords:** canine rabies, rabies education, curriculum integration, Philippines, rabies knowledge, animal bite incidence

## Abstract

As part of a province wide rabies elimination program, rabies specific information was integrated into the curriculum of all public elementary schools in Ilocos Norte, Philippines using a specifically developed teachers' manual. The rabies educational messages included rabies, animal bite prevention, bite management and responsible pet ownership and were integrated into lessons in several subjects. Four elementary schools were randomly selected and an assessment of the change in student's rabies knowledge and animal bite incidence were conducted. The study tested all students in grades 1–5 before the curriculum integration was implemented and retested these cohorts 1 year later, after implementation. Awareness of rabies was high before the implementation, likely due to the province-wide elimination campaign. However, awareness still increased significantly across all schools, and detailed knowledge of rabies increased significantly in all but one school and age cohort. Bite incidence in the 6 months prior to each survey was also recorded and the percentage of students suffering animal bites fell significantly between the two tests. The data suggested that knowledge increase correlated with decreased bite incidence in some groups but not all, suggesting a more complex relationship between knowledge acquisition and behavioral change which warrants further investigation.

## Introduction

Without community awareness and understanding of the risks, participation in rabies control measures is likely to remain too low to achieve elimination of the public health threat of rabies.

Educational interventions are commonly employed in community-based health interventions, but often their impact is assumed and not formally assessed. A few studies have shown increases in rabies-awareness and more specific knowledge related to rabies and dog bite treatment using a variety of different educational methods. These include a 1 h educational intervention for school children in Sikkim, India (1), a curriculum integration program for elementary school children in El Nido, Philippines (2), rabies education information sessions and rabies information text messages in China (3), an “edutainment” campaign for schoolchildren in Sri Lanka (4) and an awareness campaign using posters, leaflets and text messages in Azerbaijan (5). However, in most of these studies, the assessment of knowledge increases was conducted over a very short timescale (days to months), and it is unclear how long this knowledge was retained, or whether it resulted in any change in behavior regarding the disease.

Community based surveys in three provinces of the Philippines have revealed that around 5% of the population are bitten or scratched by animals each year, with incidences around 50% higher amongst those under 15 years of age (6). Between 2008 and 2016, over 1/4 of Filipino human rabies deaths were children <15 years old (7). Children often play with dogs, may not report bites to adults and because of their smaller size face a higher risk of severe dog bites and of developing rabies from a rabid animal bite than adults (8). If children can be armed with the knowledge to help reduce their risks of rabies exposure, this benefit could last throughout their lifetimes, and potentially spread to their families and others in the community.

In 2013, 92.6% of Filipino children 6–11 years old were enrolled in elementary schools (9), making it effective for interventions to reach almost all young children through these schools. Since children spend a significant amount of time in school, teachers can play a vital role in raising their awareness of health issues. One of the pioneering programs of school-based health education is the Global Health for School Initiative (GHSI) launched by the World Health Organization (WHO) in 1995. The aim of GHSI is to increase the number of “Health Promoting Schools” (schools that constantly strengthen their capacity as a healthy setting for living, learning, and working), to improve the health of students, school personnel, families, and other members of the community through schools. Evidence on the effectiveness of school-based health programs was documented in the report of the WHO School Health Technical Meeting in Bangkok, Thailand in 2015 which highlighted many health impacts including: successful early detection of vision and hearing disorders in children in Bhutan and Nepal; improved oral health in Thailand; and increasing immunization coverage in Indonesia (10).

An example of a health-based initiative in schools in the Philippines is the “Fit for School Program” which was implemented in public elementary schools to raise awareness on general health promoting behaviors. It emphasized the special role of schools which as a “second home” of children should serve as a “healthy place” where habits such as handwashing, brushing teeth with fluoride toothpaste, and deworming are institutionalized as a routine activity for children (11). The program promotes the Essential Health Care Package for schoolchildren to prevent the most prevalent diseases such as respiratory tract infection, diarrhea, soil transmitted worms, and tooth decay.

The Communities Against Rabies Exposure (CARE) Project, began in April 2012, as a collaboration between the Global Alliance for Rabies Control and the Ilocos Norte Provincial Veterinary Office, Provincial Health Office, and the Department of Education Division of Ilocos Norte. This project delivered a comprehensive dog vaccination program and an education campaign aimed at increasing awareness of the need for post exposure prophylaxis (PEP). It led to the elimination of human and animal rabies cases from Ilocos Norte by 2015 (12). In order to reach children with life-saving rabies information, the CARE Project incorporated school-based education of children on rabies, where rabies educational information was integrated into the curriculum of several subjects for all elementary grades, as trialed in other provinces (2, 13). It was hoped that imparting basic rabies knowledge at an early age would help equip children for a lifetime of practicing responsible pet ownership and preventing rabies exposures and deaths. It was anticipated that rabies curriculum integration, would also support the sustainability of the rabies control program, as the rabies information messages will be reinforced by teaching each year and may be valuable in decreasing animal bite incidence, and ultimately, eliminating rabies. The initiative was in support of the mandate of the Department of Education to integrate rabies in the national education curriculum as stipulated in the Anti-Rabies Act of 2007 (Republic Act of 9482) (14).

This study assessed the impact of a Rabies Prevention Program Manual for Grade School Curriculum Integration and Instruction in Ilocos Norte. The manual was developed in collaboration with the Ilocos Norte Department of Education, Provincial Veterinary Office and Provincial Health Office, using both qualitative and quantitative methods. A pre-test (grades 1–5) and post-test of the same cohort (grades 2–6) were conducted to measure the change in rabies-related knowledge and practices of elementary students, before and after the introduction of the manual. Further qualitative feedback on the program was gathered via focus group discussions with the teachers using the materials.

## Methods

### Site Selection

**Figure 1:**
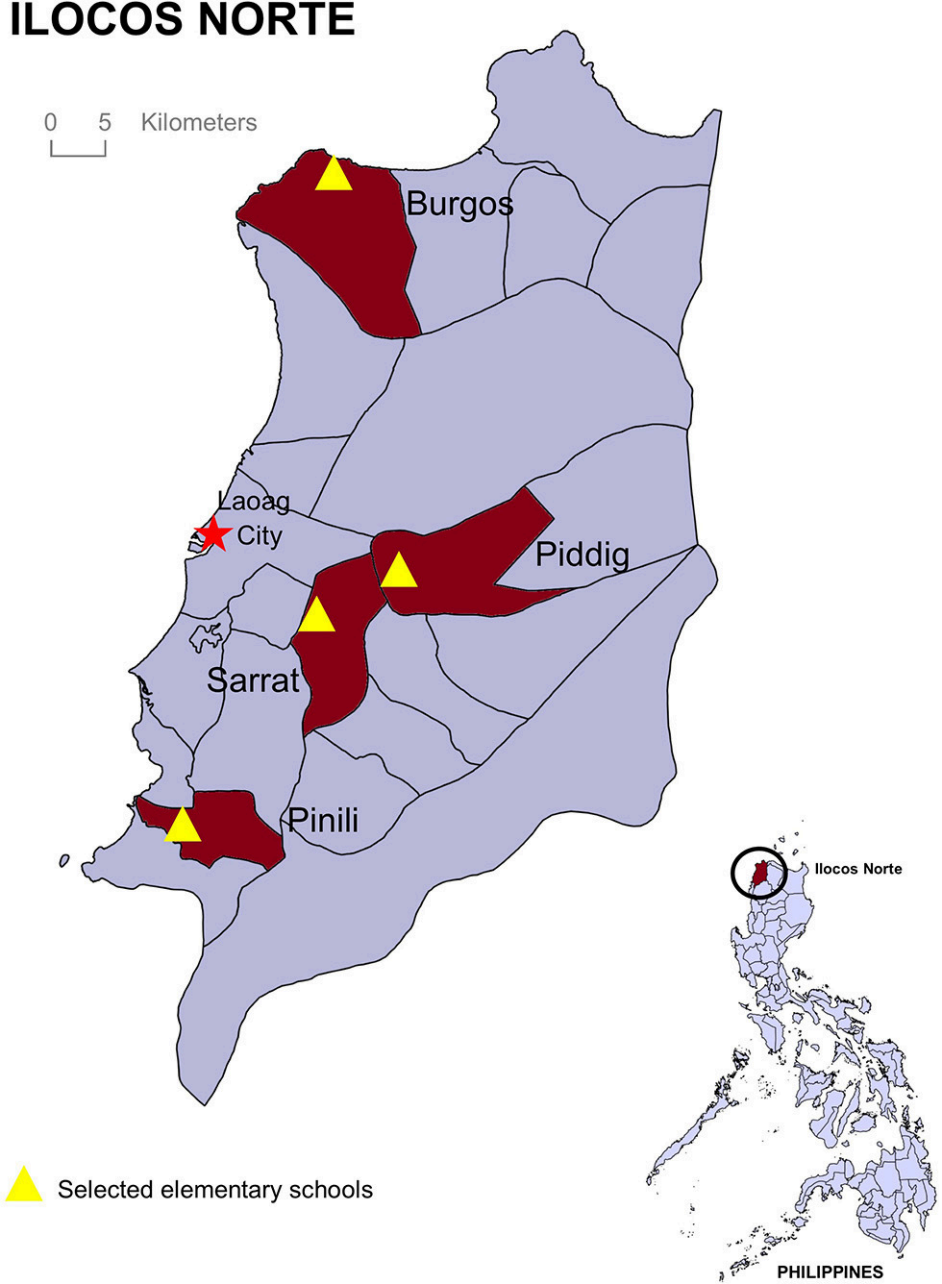
The four municipalities and the elementary schools where assessments were completed. The red star indicates the capital city of the province.

All public elementary schools in the province of Ilocos Norte province (in the far north of the country) were grouped into four geographical zones: North, South, East, and Central. Only complete public elementary schools (*n* = 286), or schools that offer levels Kindergarten to Grade 6, were included in the sampling frame. Primary public elementary schools (*n* = 58), or schools that offer only Kindergarten to Grade 4, were not included. One public elementary school was then randomly selected per zone, and these fell into the municipalities of Burgos, Piddig, Sarrat, and Pinili (see map in Figure 1). The elementary schools in Piddig and Sarrat were larger and in more urban settings close to the capital Laoag City, whilst the schools in Burgos and Pinili were smaller and in more rural municipalities.

### Integration of Rabies Education Into the Curriculum

The Ilocos Norte Rabies Prevention Program Manual on Grade School Curriculum Integration and Instruction manual was developed by the Global Alliance for Rabies Control in partnership with the Ilocos Norte Provincial Veterinary Office, Provincial Health Office, and the Department of Education Divisions of Ilocos Norte, Batac City, and Laoag City. It was adapted from a previously developed manual used in El Nido (2). The manual is comprised of modules with rabies-related lessons and examples which the teachers could integrate into lessons in Science and Health, Makabayan (Arts, Civics, Social Studies, Geography, and History), Filipino, English, and Mathematics. Lesson plans integrating information on rabies, animal bite prevention, bite management, and responsible pet ownership were designed specifically for each grade level from grade 1 to 6. For Ilocos Norte, 12 Master Teachers were involved in the workshop to develop the lesson plans included in the manual with supervision from the Ilocos Norte School Division on 6–8 May 2013.

Students from Kindergarten to Grade 6 are generally from ages 5 to 11 in the Philippines. The school year runs from June to April.

The manual was launched by 24 January 2014 and 3,390 manuals were distributed to all school divisions in Ilocos Norte with the aim for each public elementary teacher in the province to receive a curriculum manual for use from February to April and from June to October in 2014, prior to the post-tests conducted in November 2014. Table 1 shows a summary of the number of schools and manuals distributed in each of the school divisions.

**Table 1:** Number of schools and manuals distributed in each of the school divisions.

**School division**	**Number of schools**	**Number of manuals distributed**
Ilocos Norte	320	2,706
Batac City	26	233
Laoag City	34	451
Total	380	3,390

### Focus Group Discussions

In November 2014, focus group discussions were held with teachers from the 4 selected schools. Teachers from different grade levels and different subjects were invited, however, only those who were available during the dates of the FGD were able to participate. These discussions were to qualitatively assess (i) the teachers' utilization of the manual; (ii) the usefulness of the Rabies Curriculum Integration Manual in terms of its comprehensibility, applicability to the local setting, logical flow of concepts, appropriateness to each grade level, ease of incorporation into teaching; (iii) teachers' self-perceived capability in discussing rabies; (iv) whether information about other diseases should be incorporated in the grade school curriculum; (v) teachers' concerns or suggestions regarding the development, distribution and content of the manual; and (vi) whether other materials would facilitate better usage of the manual.

### Pre- and Post-testing

All students enrolled from Grades 1 to 5 in the four selected schools who were present on the scheduled dates were included in the pre-test in November 2013. Twelve months later all students in Grades 2 to 6 (present on the testing day) in the same schools were included in the post-test (Table 2). Grade 6 students were not included in the pre-test as they would not be available for post-testing, and grade 1 students not included in the post-test since the manual was not used in Kindergarten. For analysis the students were grouped into cohorts reflecting the same student group (i.e., with a post-test taken one grade higher than the pre-test it is compared to).

**Table 2:** Schools assessed and dates of implementation of the project.

**School Name**	**Municipality**	Students (% male) enrolled in grades 1–5^*^	**Pre-test date**	**Post-test date**	**Grade school Teachers in Focus Group**
Ab-Abut Elementary School	Piddig, East Ilocos Norte (urban)	136 (56.6%)	6 Nov 2013	13 Nov 2014	6
Tanap Elementary School	Burgos, North Ilocos Norte (rural)	68 (55.9%)	6 Nov 2013	13 Nov 2014	3 (+ 1 in Kindergarten)
Pandan Elementary School	Sarrat, Central Ilocos Norte (urban)	161 (60.2%)	7 Nov 2013	14 Nov 2014	5
Puzol Elementary School	Pinili, South Ilocos Norte (rural)	39 (64.1%)	7 Nov 2013	14 Nov 2014	3 (+ 1 in Kindergarten)

* *For the school year 2012-13, taken from Government of Philippines (15)*.

Two versions of a structured self-administered questionnaire were developed, one for students in Grades 1–3 and one for students in Grades 4–6 (see Figures S1A,B). The questionnaire consisted of three sections. The first part collected the student's basic demographic information. The second section consisted of questions about the respondents' sources of rabies information, any bite incidents that occurred between June and October (i.e., from the start of the school year) that year, and how that bite incident was managed. The third section included ten statements about responsible pet ownership, bite prevention, bite management and rabies, to which the respondent answered “true” or “false.” These statements differed slightly in terms of sentence structure and complexity for the two grade level groups. The questionnaires used in the pre-test and post-test was exactly the same, except for the order of statements in the third part. To check the age-appropriateness and understandability of the questions, these questionnaires were pretested informally with a small group of students of the same age group as the target students. Several of the questions were rephrased based on the results of these pretests.

The questionnaires were distributed in classrooms and the results compiled by CARE project staff, not the teachers. Younger students were assisted with recording their answers on the questionnaires by CARE project staff if necessary. Teachers were requested to not be present in the classrooms to prevent any intentional or unintentional coaching.

Comic books about a boy and his dog were given to the students as tokens for their participation. These comic books were designed for children and contained messages about rabies, responsible pet ownership, and bite management.

### Analysis

Student responses to the questionnaires were anonymous, so pre- and post-test data was not paired for individual students. However, a very high proportion of enrolled students were surveyed at each time point, thus knowledge data represent the same student group, before and after the intervention period. Data was entered in Epi Info v.3.5.4. and analyzed in Epi Info v7.2.2.6 using uncorrected Chi-squared for percentage data and using Kruskal Wallis tests for the knowledge score data. Graphs were generated using Microsoft Excel 2013.

## Results

### Qualitative Assessment of the Manual's Value From Focal Group Discussions With Teachers

Focus Group discussions (FGDs) were conducted in November 2014 in each of the four different schools, led by CARE project staff, with a total of 19 teacher–participants from the 4 schools assessed (Table 1). The number of teachers attending the four focus group discussions is given in Table 2. Only participants from the elementary school in Burgos started using the manual regularly in February. Due to delays in distribution, participants from the other schools were only able to use it from the start of the new school year. The discussions revealed that teachers were not always following the lesson guides and flow of activities exactly as suggested in the manual, but integrated information from the manual into their existing teaching plans where they fitted best.

The manual was commended for its ease of use, comprehensibility, relevance, applicability to the local setting, and appropriateness to each grade level. Some participants added that they could use some of the first graders' lesson guides in teaching kindergarteners. Teachers reported that their students memorized the subject content rapidly because of the short-structured messages and its repetition of information on rabies. Some also shared how students found the stories in the manual entertaining and funny and enjoyed borrowing and reading the manual by themselves. Most of the book's content was judged self-explanatory. Teachers reported that the information had proved useful for first aid advice in instances when their students were bitten.

Teachers reported that the content in the manual had changed the children's notion that rabies can only be transmitted through dog bites, and that the content had been used for first aid advice in instances when their students were bitten. There were also suggestions which could be used to improve future editions of the manual, including dividing the materials for different grades into separate manuals, clarifying some issues where conflicting information from other sources could cause confusion, adding more color pictures, providing more evaluation materials such as pre-tests or post-tests for each lesson, providing more supporting materials for students such as student textbooks, posters, rabies-related videos, CDs of songs included in the manual or comics, and correcting some minor typographical errors. Teachers suggested that educational materials for the students in the local dialect would be useful for younger students, but that the available Filipino or English versions should also be used.

After receiving the manual, the majority of the participants felt confident about their knowledge of rabies and were comfortable with discussing it with their students, but also referred to other sources of rabies information such as textbooks from barangay (village) health centers or other schools and the internet. They indicated that they would be willing to learn more if the developers of the manual had additional information about rabies. Specific detailed questions about rabies not directly addressed by the manual (such as the incubation period of the rabies virus in humans; the exact regimen of rabies PEP and whether puppies are more rabid than mature dogs) were answered by the session facilitators. Suggestions for how to discuss the rabies information with parents of the students were also brought up, and suggestions of seminars on responsible dog ownership for parents to attend with their students.

Participants from all schools believed that information about the control of mosquito-related diseases (i.e., dengue, malaria, and chikungunya), ebola, and leptospirosis would be beneficial to add to the curriculum using a similar manual format.

### Survey of Students

A total of 364 students (grades 1–5) from the 4 schools were included in the pre-test in November 2013 while 335 students (grades 2–6) were included in the post-test in November 2014 (Table 3).

**Table 3:** Demographic characteristics of elementary students included in the pre-test and post-test.

**Characteristics**	**Pre-test November 2013**		**Post-test November 2014**	
	**(grades 1–5**, ***n*** **=** **364)**		**(grades 2–6**, ***n*** **=** **335)**	
**Municipality: school name**	***n***	**%**	***n***	**%**
Burgos: Tanap Elem School	52	14.3	52	15.5
Piddig: Ab-Abut Elem School	133	36.5	122	36.4
Pinili: Puzol Elem School	34	9.3	37	11.0
Sarrat: Pandan Elem School	145	39.8	124	37.0
**GENDER**				
Female	155	42.6	152	45.4
Male	209	57.4	183	54.6
**COHORT**				
1—Grade 1/2	78 (6.1)	21.4	76 (7.2)	22.7
2—Grade 2/3	81 (7.4)	22.3	64 (8.4)	19.1
3—Grade 3/4	66 (8.5)	18.1	63 (9.4)	18.8
4—Grade 4/5	70 (9.8)	19.2	63 (10.7)	18.8
5—Grade 5/6	69 (10.4)	19.0	69 (11.4)	20.6
**PET OWNER**				
Yes	308	84.6	282	84.2
No	56	15.4	53	15.8

*The numbers inside the parentheses denote the mean age of the students in that particular grade level*.

Demographic characteristics of the students are shown in Table 3. The majority of the students attended the larger urban elementary schools in Piddig and Sarrat, both located near the main town (Laoag City) with fewer attending the two smaller schools located in more rural areas in the north and south of the province (Figure 1, Table 1). Slightly more than half of the students in both tests were male (average 55%) closely reflecting the student cohort overall (Tables 1, 2). The ages of the students during the pre-test ranged from 5 to 14 years, while the ages of those in the post-test ranged from 6 to 15 years old. Around 85% of the students were pet owners at the time of both tests (Table 3).

### Students' Awareness and Knowledge About Rabies

**Figure 2:**
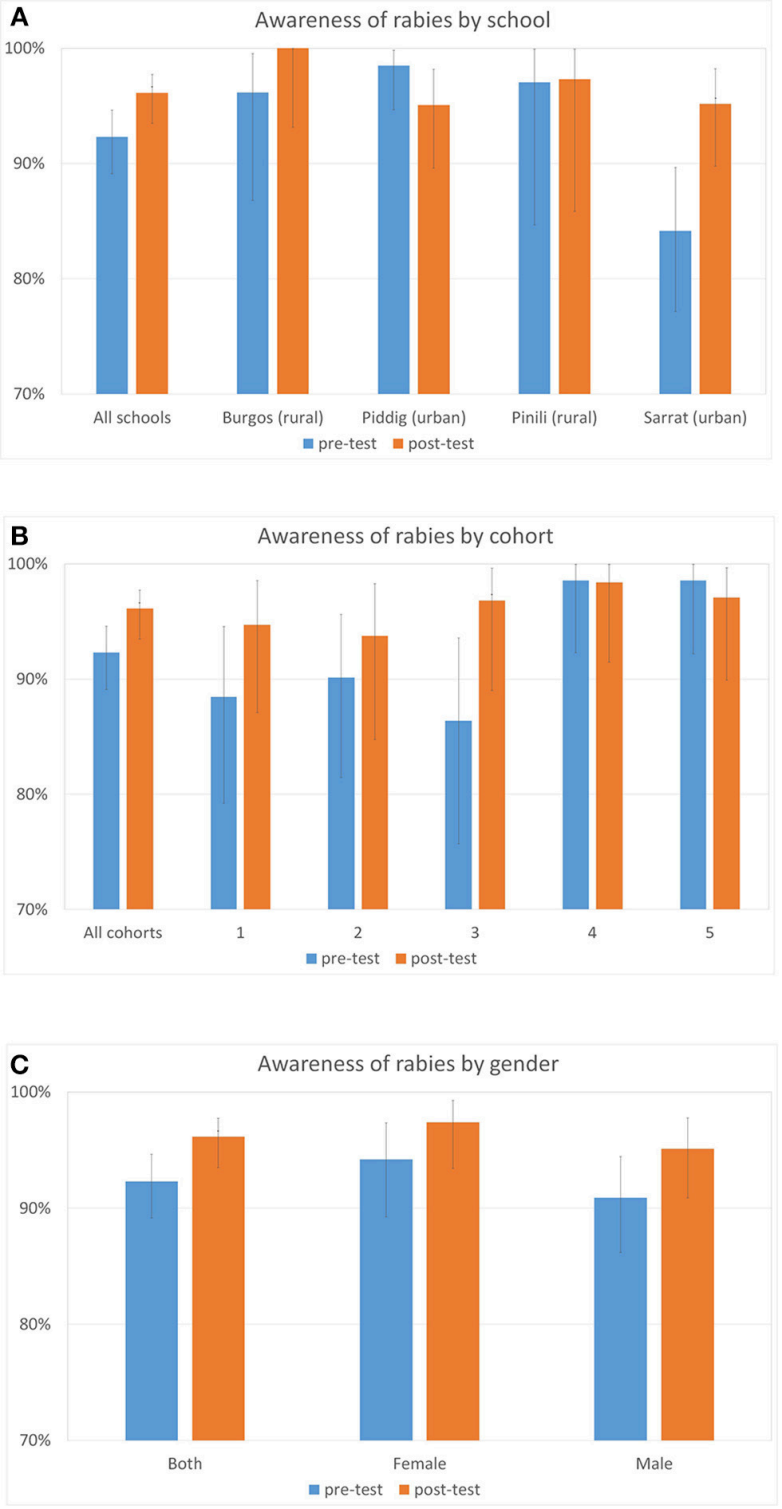
Percentage of students who had heard about rabies in the pre- and post-tests by school **(A)** by cohort **(B)** and by gender **(C)**. Error bars are 95% CIs, and asterisks denote significant differences from pre- to post- test.

Awareness of rabies was high in the pre-test, across all schools and cohorts. Across all students, 92.3% answered that they had heard about rabies, and this increased to 96.1% for the post-test, a significant rise (Figure 2A, chi-sq 1df = 4.59, *p* = 0.032). There was variation between schools, but no consistent difference between urban and rural schools, and only the school in Sarrat showed a significant change, as awareness was lowest in the pre-test here (Figure 2A, chi-sq 1df = 8.44, *p* = 0.004). Improvements in the percentage of students who had heard of rabies were only seen in the younger cohorts, who were less likely to have heard of rabies at the pre-test stage, and was only significant for cohort 3 (Figure 2B, chi-sq 1df = 4.52, *p* = 0.033). Male students had a lower awareness of rabies than females, but awareness rose in the post-test for both genders, though not significantly (Figure 2C).

**Figure 3:**
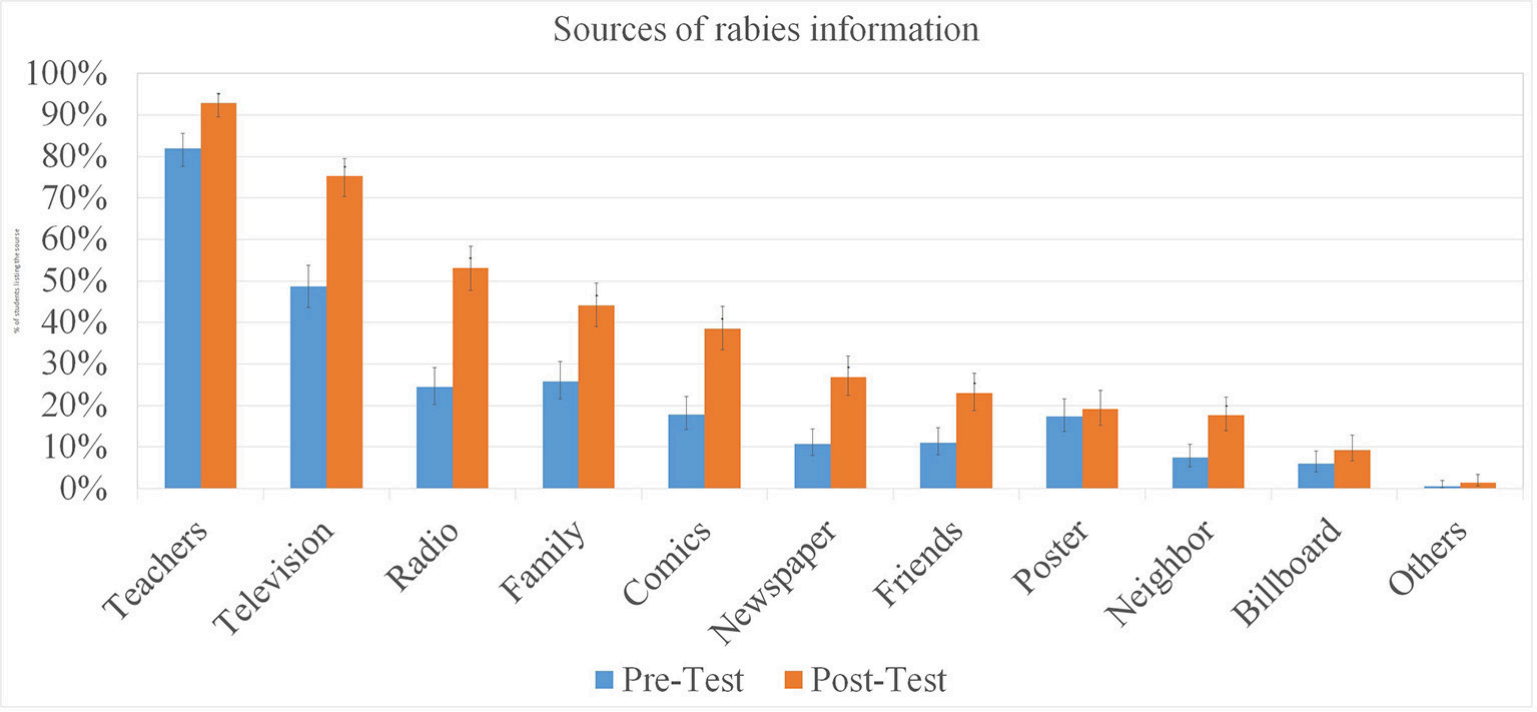
Sources of rabies information for elementary students.

Figure 3 shows the students' sources of information on rabies. The majority of the respondents reported to have heard of rabies from their teachers (pre-test 81.9%; post-test 92.8%), television (pre-test 48.6%; post-test: 75.2%), radio (pre-test: 19. post-test:52%) and family members (pre-test:25.8%; post-test:44.2%). Almost 40% of the students included in the post-test remembered learning about rabies in the GARC comic book which were given to the students during the pre-test.

Specific aspects of rabies knowledge were assessed by a set of statements covering responsible pet ownership, bite prevention, bite management, and rabies transmission, which students were asked to state as true or false. Simpler language for these statements was used for grades 1–3 compared to grades 4–6 (see Figures S1A,B). The proportion of students correctly answering each statement is given in Table S1.

Overall, the proportion of students that correctly answered all of the questions in each rabies knowledge theme increased between the pre- and post-tests in all areas except the rabies disease and prevention category which also started off low (Table 4). Looking at the detailed results in Table S1 it is clear that this was due to answers to Question 9 on the fatality of rabies (RAB2). This effect was seen across all schools, but only in the older cohorts. Understanding of the fatality of rabies disease improved in the lower grades, but fell in the higher grades. It is suspected that the wording of the statement for the older grades (“Rabies is preventable but not curable”) was too complex and confused the students. Knowledge of rabies transmission from animals to humans, and that rabies can be prevented by vaccination improved between pre- and post-tests for all school and across all cohorts (Table S1).

**Table 4:** Summary data on rabies knowledge scores.

**Knowledge area (code)**	**Number (percentage) children with perfect score in pre-test**	**Number (percentage) children with perfect score in post-test**
Responsible pet ownership (RPO)	227 (62.4%)	277 (82.7%)
Bite prevention (PREV)	271 (74.5%)	283 (84.5%)
Bite management (BITE)	268 (73.6%)	290 (86.6%)
Rabies disease and prevention (RAB)	161 (44.2%)	130 (38.8%)

Further analysis of knowledge scores was completed by excluding question 9 and summing the total number of correct answers to the remaining (nine) questions.

**Figure 4:**
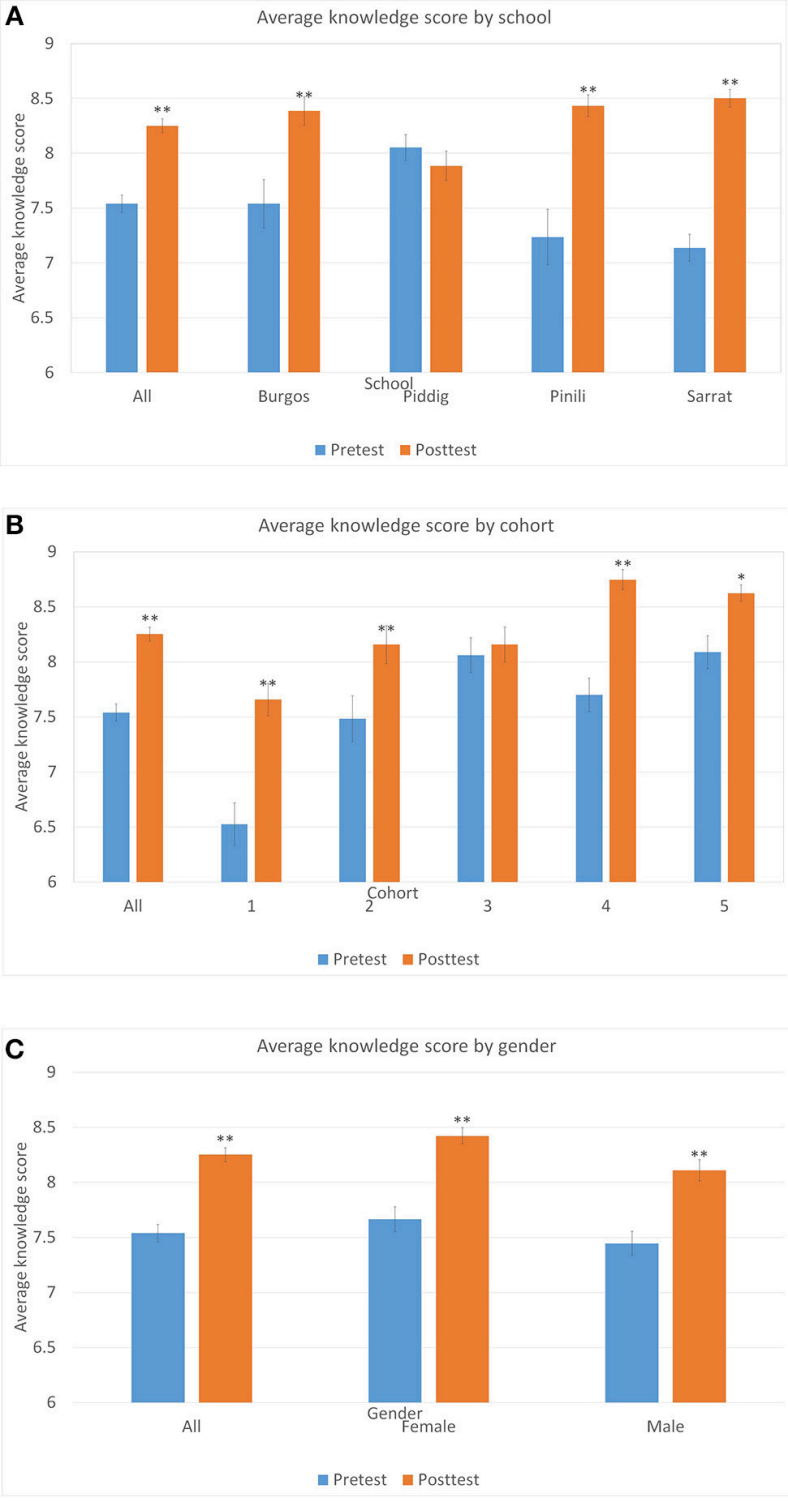
Average knowledge score (maximum score of 9) by school **(A)**, cohort **(B)**, and gender **(C)**. Error bars are standard errors, and asterisks denote significant changes ^**^p < 0.01, ^*^p < 0.05.

The mean score across all students rose from 7.54 to 8.25, a significant increase (Figure 4, Kruskal–Wallis H, 1df = 51.20, *p* = 0.000). Knowledge increased significantly for three out of the four schools (Figure 4A). As anticipated, the students' average knowledge scores in the pretest rose amongst older cohorts, but in all cohorts but one there was still a significant increase in knowledge after the intervention (Figure 4B). The mean score rose significantly for both genders (Figure 4C).

### Animal Bite Incidents and Health Seeking Behavior

**Figure 5:**
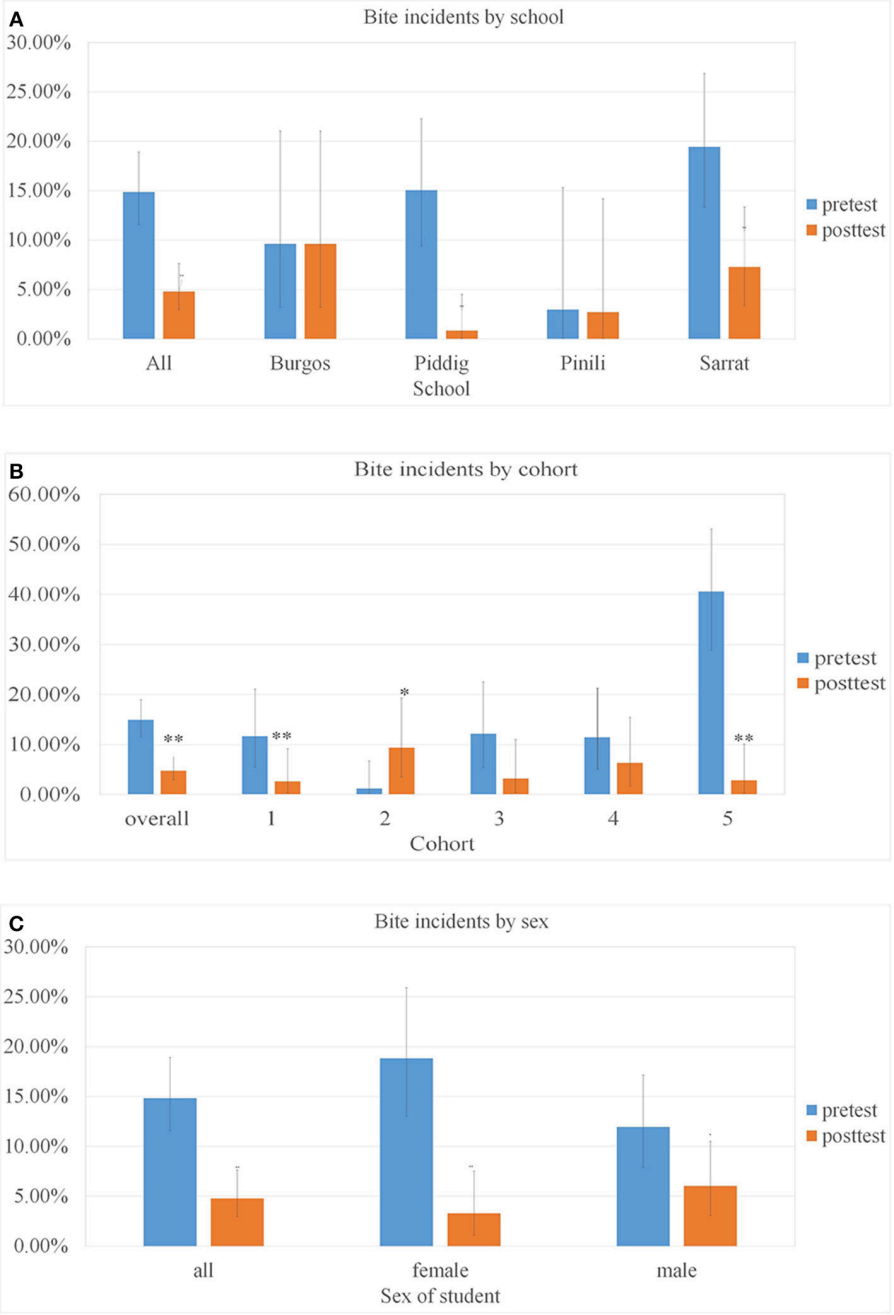
The percentage of students reporting a bite incident from June to October priort to the survey dates, by school **(A)**, cohort **(B)**, and gender **(C)**. Error bars are 95% CIs and asterisks denote significant changes ^**^p < 0.01, ^*^p < 0.05.

The number of students reporting a history of animal bite incident (dog or cat) between June and October prior to the pre-test was 54 (14.9% of 364, Figure 5). Higher percentages of students from the urban schools (in Piddig and Sarrat) reported bites compared to the rural schools in Burgos and Pinili (Figure 5A), and these were concentrated in the oldest age cohort (Figure 5B).

Only 16 students (4.8% of 335) across all schools reported bite incidents for the same time period prior to the post-test, representing a significant drop overall (chi-sq 1df = 19.70, *p* < 0.001). Figure 5 shows the percentage of students involved in such incidents by school, by cohort and by gender. The number of students reporting bites in some of these categories is very small, but bite incidence fell significantly in the two urban schools, but did not change in the rural schools, fell most significantly in the oldest age cohort and amongst female students (Figure 5).

Amongst students reporting bites in the pre-test, 25 out of 54 (46.2%) were female, and amongst those reporting bites at the post-test, 5 out of 16 (31.2%) were female, a change that was significant (chi sq 1df = 18.71, *p* < 0.001).

**Figure 6:**
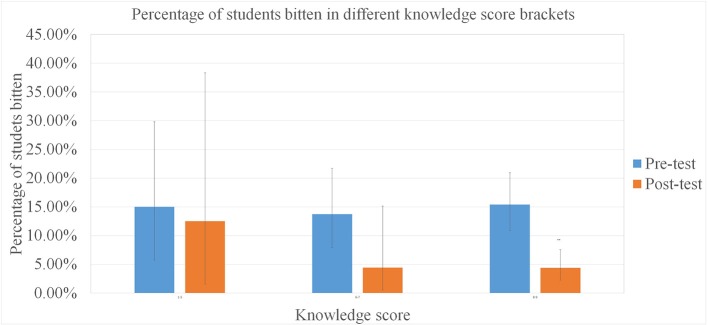
The percentage of students reporting a bite incident from June to October prior to the survey dates, by rabies knowledge score bracket. Error bars are 95% CIs and asterisks denote significant changes ^**^p < 0.01, ^*^p < 0.05.

No simple relationship was found between the bite incidence and the knowledge level, suggesting that knowledge about rabies may not relate directly to changes in behavior affecting rabies risk. Students with a high level of knowledge the pre-test still suffered high bite incidences, yet in the post-test, it appeared that higher levels of knowledge correlated with lower bite incidences (Figure 6).

Of the students reporting bites prior to the pre-test, data on health seeking behavior was available for 48. Of these, 32 (66.6%) sought medical treatment at a hospital or bite clinic. Of the 16 students reporting bites prior to the post-test survey, all answered the question, and 9 (56.2%) sought medical treatment, an insignificant change (chi-sq 1df = 0.57, *p* = 0.45).

## Discussion

The reception of the curriculum integration manual by the teachers in this study was very positive and most felt comfortable teaching about rabies and answering questions from their students. Although there were suggestions for improvements, and additional material that could be supplied, the lesson plans were deemed valuable and easily integrated into teaching. In some cases, teachers adapted how they used the information in the manual to better fit their teaching, deviating from the lesson plans suggested in the manual. There were clear examples where the rabies information had been put into practice by teachers, such as in the event of an animal biting incident, and supporting children and their families with rabies control guidance, demonstrating the role teachers have in disseminating information to the wider community beyond their students.

Several factors increase the difficulty of monitoring the impact of this educational intervention. The curriculum integration program was part of a larger rabies control program, and students were exposed to many different sources of information on rabies. Community awareness was also being increased through mass dog vaccination campaigns. A Media Awareness Workshop was conducted in 2013 for media practitioners in the province to encourage reporting accurate messages on rabies especially, on animal bite management and mandatory vaccination of dogs against rabies. The Philippine Information Agency of Ilocos Norte who is the lead government agency for increasing awareness through different channels is a member of the Ilocos Norte Provincial Rabies and Control Committee which made it possible for rabies to be featured regularly in the tri-media (television, newspaper, and radio) (12).

Students reported many different sources of rabies information in both the pre-test and post-test, so it appears that students were acquiring information about rabies from multiple sources. This attested to the efforts of the province-wide elimination campaign to reach the community through many different information and education campaigns. However, more students reported teachers as a source of rabies information than any other source, suggesting that knowledge acquired in schools had a strong influence over their understanding. It is also likely that whilst other information sources could account for the high awareness of rabies amongst students in the pre-test, they did not provide them with the more detailed understanding of rabies transmission and prevention afforded by the curriculum integration program. This effect was suggested to be true in intervention “Rabies Edutainment 4 Kids” (an education-entertainment campaign) in a rural area of Sri Lanka in 2009 (4).

The curriculum integration manual was supplied and expected to be used across all elementary schools in Ilocos Norte at the same timepoint, thus it was not possible to assess the knowledge increases in an appropriate control group of students. It was noted in the focus group discussions that some rabies information was already being taught in schools prior to the intervention, which would account for the relatively high awareness of rabies and the percentage of students citing teachers as a source of this information in the pre-test. Finally, the focus group discussions revealed that the way in which materials from the manual were used differed from the suggested methods in some cases. All teachers reported that they had integrated some of the manual into their teaching, but because of the differences in implementation, it is likely that some students had the messages reinforced more than others. However, given the constraints of a program rolled out across all school in a province, complete uniformity in teaching practices should perhaps not be expected, and this assessment might therefore be considered more realistic than a smaller intervention with more controlled intervention.

There was evidence that one particular question was phrased too poorly for the students to be able to understand, one which required them to distinguish between “preventable” and “curable.” This distinction is critical for rabies, but very often misunderstood and phrasing around this issue may need further evaluation in future educational materials.

Despite these limitations, the pre- and post-test results demonstrated that overall, students' awareness of rabies increased. The more detailed analyses revealed that specific rabies knowledge increased significantly in all but one school, all but one cohort and amongst students of both genders. It is possible that some of the differences observed across schools and cohorts could be explained by non-uniform implementation of the manual by teachers, but it is not possible to explore this from the data available. The only cohort of students that showed a non-significant increase in rabies knowledge was cohort 3 who were tested with the simpler questionnaire in the pre-test, but the slightly more complex questionnaire in the post-test.

In general, rabies educational interventions that increase understanding and knowledge of disease are assumed to lead to behavioral change that helps to reduce the risk of disease. As far as we are aware only two previous rabies educational interventions also attempted to assess behavioral changes resulting from increased knowledge. No significant impact on bite incidence was reported in El Nido (2), but self-reported dog vaccination rates did increase in Azerbaijan (5).

In the current study there was a significant decrease in the proportion of students who had a history of animal bite from June to October in the post-test, compared to the same months a year earlier as reported in the pre-test. Despite sample sizes of bites being small, the results suggest that a behavioral change in the way students interacted with dogs may have occurred over the intervention year. The overall data suggest that increased rabies knowledge correlated with lower bite incidence, and female students showed higher increases in knowledge and larger decreases in bite incidences. However, two aspects of the data suggest that there may not be a simple relationship between increased knowledge and behavioral change. The largest drops in bite incidence between the pre- and post-test occurred in the school in Piddig where rabies specific knowledge did not significantly increase, and in the pre-test students with higher rabies knowledge scores did not report lower bite incidences. Higher knowledge scores in the post-test did however correspond to lowered bite incidences. The sample sizes in this study are too small to be certain, but it is possible that some other aspect of understanding, not captured by the pre- and post-test questions contributed to the reduction in bite incidence. Further investigations with larger sample sizes would be needed to investigate this more thoroughly.

Comparing two urban (Piddig and Sarrat) and two rural (Burgos and Pinili) schools allowed us to reveal a higher rate of dog bite exposures for students living in urban environments. Despite this, awareness of rabies was high in both settings, with the age of the students having a more important effect on awareness and detailed knowledge about rabies. Irrespective of the precise mechanism causing it, the significant reduction of bite incidence in urban environments (where the higher bite incidences were reported in the pre-test) could represent a significant reduction in the risk of rabies for elementary students. It would be of interest to repeat the survey with a larger cohort of rural students to see if there is a detectable impact of the increase in knowledge on bite occurrence.

The interval between pre- and post-test was relatively short, and the distribution process of the manual to all teachers in Ilocos Norte was not very efficient. At a consultation meeting with teachers held in May, many participants had still not received their manuals. Together with the variation in the application of the teacher manual across schools, this may have reduced the impact of the curriculum integration. A more long term follow up of knowledge scores across more schools would enable an assessment of whether repeated exposure to the curriculum over successive years has a stronger impact. Such a larger sample size would also enable a more rigorous assessment of whether a change in health seeking behavior following a bite incident was occurring amongst the families of elementary school students.

The findings of this study, however, show promise in the use of a rabies curriculum integration manual in raising rabies awareness in both children and teachers. After discussions of this study's findings, the Department of Education of Ilocos Region (Region I) officially endorsed the use of the rabies education curriculum manual in public elementary schools in Ilocos Norte on 13th February 2015. A total of 395 teachers from 380 public elementary schools across all three school divisions in Ilocos Norte were given additional training in the use of the manual between February and April 2015. These participants were assigned as rabies teacher-coordinators for their respective schools and were tasked to share the information on rabies education to their fellow teachers and monitor the use of the manual.

## Ethics Statement

All CARE project activities in Ilocos Norte were conducted by the Provincial Veterinary, Health and Education offices under the implementation of the government's rabies elimination program, and as such did not undergo a separate ethics review. All services provided were bound by Republic Act 9482, Anti-Rabies Act of 2007, Republic Act 6713 Code of Conduct and Ethical Standards for Public Officials and Employees, and Republic Act 9268, The Philippine Veterinary Medicine Act of 2004, and all assessments of progress were conducted in full collaboration with the offices mentioned above. Students were asked if they agreed to participate in the pre- and post-tests and were assured that their participation (or non-participation) and test scores will not affect their grades.

## Author Contributions

JM, LV, and EM were involved in the design, development, and distribution of the manual. AA, EM, and DL developed the test questionnaires. Pre- and post-tests were facilitated by JM, LV, AA, EM, and DL. Data were curated and analyzed by LT, AA, EM, and DL. LT, SJ, AA, EM, DL, LV, and JM contributed to the writing of the manuscript and all authors reviewed it for accuracy.

### Conflict of Interest Statement

The authors declare that the research was conducted in the absence of any commercial or financial relationships that could be construed as a potential conflict of interest.
