# Supplementary material for: Impact of Integrating Rabies Education Into the Curriculum of Public Elementary Schools in Ilocos Norte, Philippines on Rabies Knowledge, and Animal Bite Incidence
Source: Front Public Health. 2019 May 24;7:119. doi: 10.3389/fpubh.2019.00119 (PMC6543910; doi:10.3389/fpubh.2019.00119)
Supplement: Supplementary file 1 [file Data_Sheet_1.docx]

**Supplementary Data**

## **Table S1. Proportion of students answering each question correctly by cohort and by school**

Cells shaded green represent at least a 5% improvement in the post test scores, cells shaded red represent at least a 5% decline compared to pretest scores.

|  |  |  | **Proportion of students with correct answer  by cohort** | | | | | **Proportion of students with correct answer  by school** | | | |
| --- | --- | --- | --- | --- | --- | --- | --- | --- | --- | --- | --- |
| Code | Question number and Topic | **Cohort** | **1** | **2** | **3** | **4** | **5** | **Burgos** | **Piddig** | **Pinili** | **Sarrat** |
| RPO 1 | Q1. Dog anti-rabies vaccination | pre | 89.7% | 95.1% | 93.9% | 92.9% | 92.8% | 90.4% | 94.7% | 85.3% | 93.8% |
|  |  | **post** | **100.0%** | **93.8%** | **95.2%** | **98.4%** | **100.0%** | **100.0%** | **94.3%** | **100.0%** | **99.2%** |
| RPO 2 | Q2. Feeding of pet dogs | pre | 51.3% | 45.7% | 93.9% | 88.6% | 94.2% | 61.5% | 93.2% | 67.6% | 60.0% |
|  |  | **post** | **69.7%** | **96.9%** | **90.5%** | **100.0%** | **100.0%** | **100.0%** | **85.2%** | **94.6%** | **91.1%** |
| RPO 3 | Q3. Confining of pet dogs | pre | 70.5% | 92.6% | 92.4% | 75.7% | 88.4% | 90.4% | 91.7% | 82.4% | 74.5% |
|  |  | **post** | **86.8%** | **87.5%** | **84.1%** | **98.4%** | **95.7%** | **92.3%** | **85.2%** | **97.3%** | **92.7%** |
| PREV 1 | Q4. Appropriate time to play with dogs / Reading dog behaviour | pre | 82.1% | 97.5% | 95.5% | 60.0% | 75.4% | 90.4% | 91.7% | 73.5% | 73.1% |
|  |  | **post** | **89.5%** | **92.2%** | **87.3%** | **92.1%** | **81.2%** | **98.1%** | **87.7%** | **70.3%** | **90.3%** |
| PREV 2 | Q5. Dealing with stray dogs | pre | 84.6% | 81.5% | 95.5% | 87.1% | 91.3% | 94.2% | 91.0% | 91.2% | 81.4% |
|  |  | **post** | **96.1%** | **93.8%** | **90.5%** | **92.1%** | **95.7%** | **96.2%** | **87.7%** | **100.0%** | **96.8%** |
| BITE 1 | Q6. First aid for dog bites | pre | 89.7% | 88.9% | 87.9% | 84.3% | 92.8% | 92.3% | 93.2% | 76.5% | 86.2% |
|  |  | **post** | **90.8%** | **95.3%** | **93.7%** | **96.8%** | **92.8%** | **94.2%** | **91.8%** | **100.0%** | **93.5%** |
| BITE 2 | Q7. Seeking an adult’s help in case of dog bite | pre | 53.8% | 88.9% | 89.4% | 92.9% | 88.4% | 69.2% | 89.5% | 88.2% | 78.6% |
|  |  | **post** | **86.8%** | **89.1%** | **85.7%** | **98.4%** | **97.1%** | **94.2%** | **85.2%** | **97.3%** | **94.4%** |
| RAB 1 | Q8. Rabies transmission from animal to human | pre | 67.9% | 71.6% | 66.7% | 91.4% | 91.3% | 82.7% | 75.9% | 64.7% | 80.0% |
|  |  | **post** | **63.2%** | **85.9%** | **92.1%** | **98.4%** | **100.0%** | **75.0%** | **85.2%** | **83.8%** | **95.2%** |
| RAB 2 | Q9. Fatality of rabies | pre | 60.3% | 75.3% | 84.8% | 21.4% | 53.6% | 75.0% | 66.2% | 52.9% | 49.0% |
|  |  | **post** | **80.3%** | **89.1%** | **41.3%** | **14.3%** | **37.7%** | **36.5%** | **69.7%** | **45.9%** | **46.8%** |
| RAB 3 | Q10. Rabies prevention | pre | 62.8% | 86.4% | 90.9% | 97.1% | 94.2% | 82.7% | 84.2% | 94.1% | 86.2% |
|  |  | **post** | **82.9%** | **81.3%** | **96.8%** | **100.0%** | **100.0%** | **88.5%** | **86.1%** | **100.0%** | **96.8%** |
