# Supplementary material for: Impact of Integrating Rabies Education Into the Curriculum of Public Elementary Schools in Ilocos Norte, Philippines on Rabies Knowledge, and Animal Bite Incidence
Source: Front Public Health. 2019 May 24;7:119. doi: 10.3389/fpubh.2019.00119 (PMC6543910; doi:10.3389/fpubh.2019.00119)

# Supplementary information

## Figure S1A. Test questionnaire for Grades 1 to 3 students. Tick marks (✓) indicate the correct answers for the knowledge questions

✓

✓

✓

✓

✓

✓

✓

✓

✓

✓


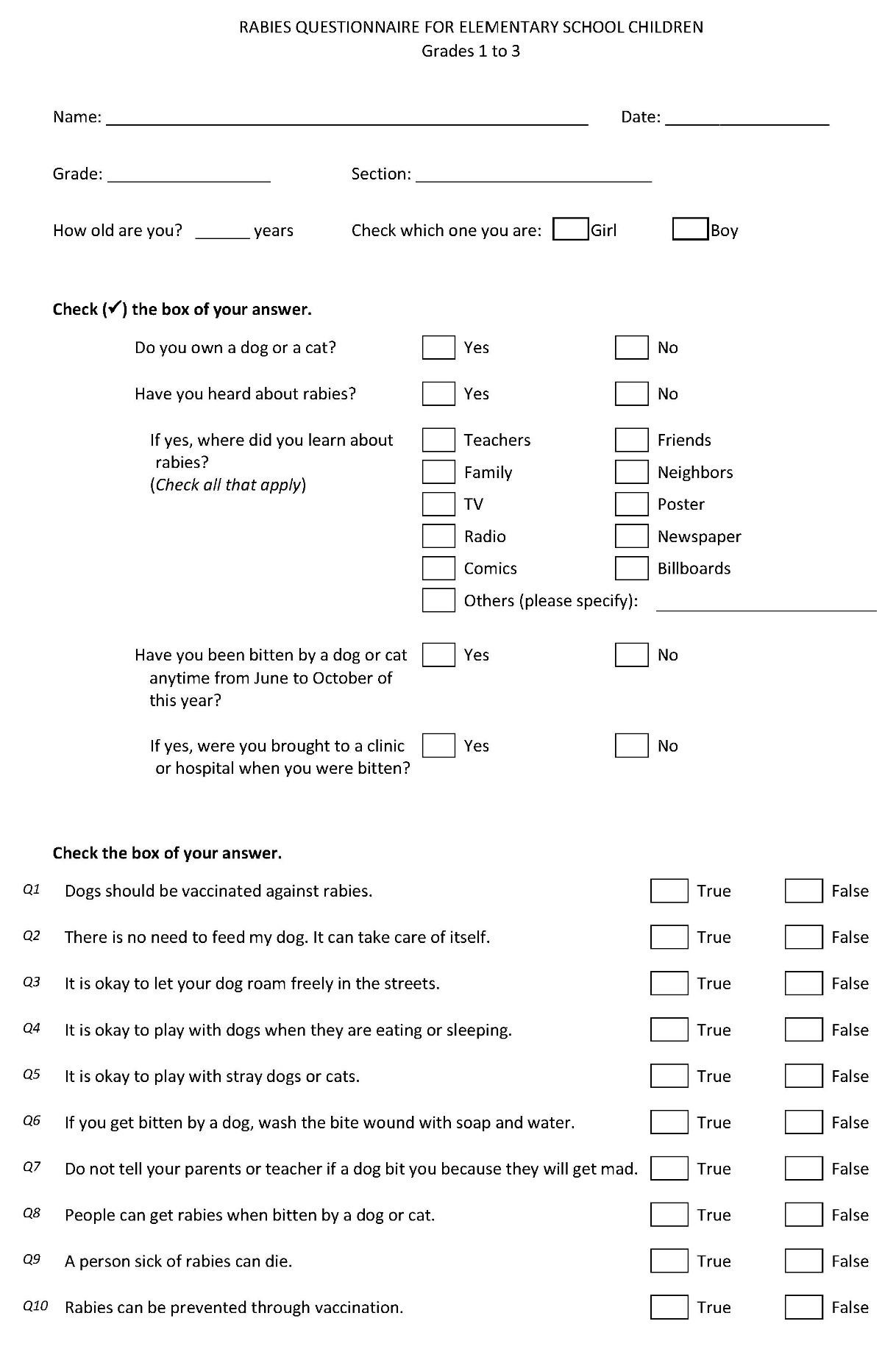


## Figure S1B. Test questionnaire for Grades 4 to 6 students. Tick marks (✓) indicate the correct answers for the knowledge questions

✓

✓

✓

✓

✓

✓

✓

✓

✓

✓


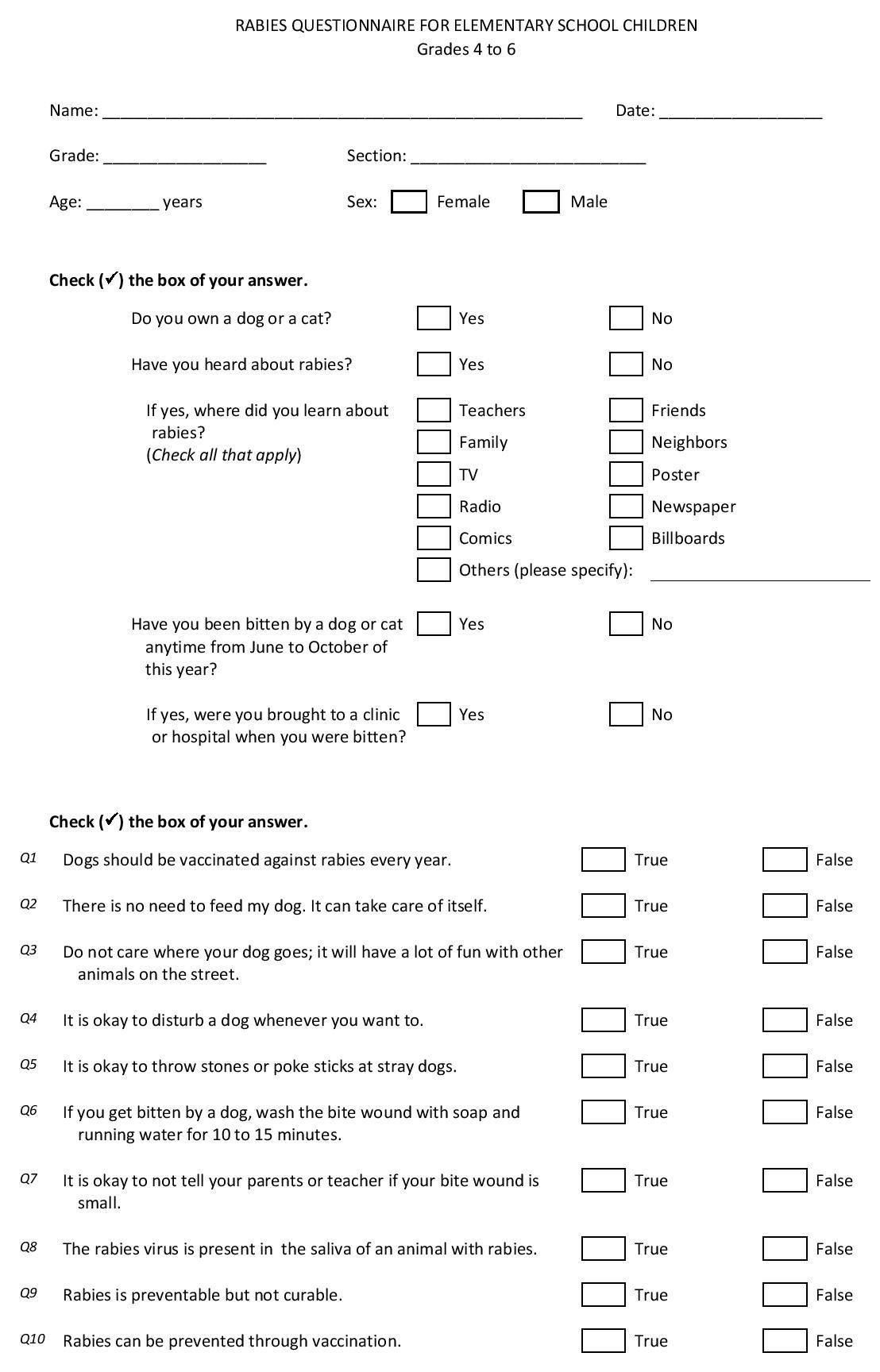

Supplement: Supplementary file 2 [file Data_Sheet_2.docx]
